# A fatal case of mitochondrial DNA depletion syndrome with novel compound heterozygous variants in the deoxyguanosine kinase gene

**DOI:** 10.18632/oncotarget.20905

**Published:** 2017-09-15

**Authors:** Weiyuan Fang, Peng Song, Xinbao Xie, Jianshe Wang, Yi Lu, Gang Li, Kuerbanjiang Abuduxikuer

**Affiliations:** ^1^ The Center for Pediatric Liver Disease, Children's Hospital of Fudan University, Shanghai 201102, China; ^2^ Advanced Training Program, Children's Hospital of Fudan University, Shanghai 201102, China; ^3^ Department of Infectious Diseases, Tangshan Maternal and Children Health Hospital, Tangshan City, Hebei Province 063000, China; ^4^ Institute of Pediatrics, Children's Hospital of Fudan University, Shanghai 201102, China

**Keywords:** mitochondrial DNA depletion syndrome (MDS), deoxyguanosine kinase (DGUOK)

## Abstract

The deoxyguanosine kinase (DGUOK) gene controls mitochondrial DNA (mtDNA) maintenance, and variation in the gene can alter or abolish the anabolism of mitochondrial deoxyribonucleotides. A Chinese female infant, whose symptoms included weight stagnation, jaundice, hypoglycemia, coagulation disorders, abnormal liver function, and multiple abnormal signals in the brain, died at about 10 months old. Genetic testing revealed a compound heterozygote of alleles c.128T>C (p.I43T) and c.313C>T (p.R105^*^) of the DGUOK gene. c.128T>C (p.I43T) is a novel variant located in exon 1 (NM_080916) in the first beta sheet of DGUOK. Her mother was an allele c.313C>T (p.R105^*^) heterozygote, which is located in DGUOK exon 2 (NM_080916) between the third and fourth alpha helixes. c.313C>T (p.R105^*^) is predicted to result in a 173 amino acid residue truncation at the C terminus of DGUOK. There are as many as 112 infantile mtDNA depletion syndrome (MDS) cases in the literature related to DGUOK gene variants. These variants include missense mutations, nucleotide deletion, nucleotide insertion, and nucleotide duplication. Integrated data showed that mutations affected both conserved and non-conserved DGUOK amino acids and are associated with patient deaths.

## INTRODUCTION

Mitochondrial DNA (mtDNA) is a 16.6 kb circular double-stranded DNA encoding the protein subunits of mitochondrial respiratory chain complexes I, III, IV, and V and which functions in energy production [1, 2]. All of the mtDNA replication, transcription factors, and the primary components of the mitochondrial translation machinery are encoded by nuclear genes [1, 2]. Mutations in nuclear genes that function in either mitochondrial deoxyribonucleoside triphosphate (dNTP) synthesis or mtDNA replication can lead to a severe reduction in cellular mtDNA content in affected organs, which is termed mtDNA depletion syndrome (MDS) [1, 2]. Thymidine kinase 2, adenosine diphosphate-forming succinyl CoA ligase beta subunit, guanosine diphosphate-forming succinyl CoA ligase alpha subunit, ribonucleotide reductase M2 B subunit, thymidine phosphorylase, and deoxyguanosine kinase (DGUOK) encode proteins that maintain the mitochondrial dNTP pool, and mutations in any of these genes can result in mtDNA depletion [2]. DNA polymerase gamma and Twinkle are essential for mtDNA replication, and mutations in these genes can result in inadequate mtDNA synthesis [2].

Clinically, MDS is classified into myopathic, encephalomyopathic, neurogastrointestinal, and hepatocerebral forms that are associated with mutations in the above genes [2]. Of the four forms of MDS, hepatocerebral MDS is associated with DGUOK gene mutations and occurs during the neonatal period [2–31]. The common clinical features of hepatocerebral MDS include hepatic dysfunction, psychomotor delay, hypotonia, rotary nystagmus developing into opsoclonus, lactic acidosis, and hypoglycemia [2–31]. Here, we report a fatal case of MDS with a novel compound heterozygous mutation in the DGUOK gene as well as a systemic literature review.

## RESULTS

### Clinical findings

Physical examination at 9 months and 4 days of age revealed slight jaundice, with a head circumference, chest circumference, height, weight, temperature, heart rate, and respiration rate of 40.0 cm, 41.0 cm, 69.0 cm, 7.0 kg, 37.2^°^C, 134 beats per minute, and 32 times per minute, respectively (Table 1). No obvious psychomotor delay, hypotonia, or rotary nystagmus was observed. Visual inspection and palpation found no obvious abnormalities except for splenomegaly.

**Table 1:** Clinical and laboratory findings

*Personal history*	
Age	9 months and 4 days
Gravidity (G) and Parity (P)	G3P1
Gestation	37 weeks+2days
Birth weight (g)	2,550
Birth history	Breech position caesarean section
*Family history*	
Pregnancy history of mother	1-0-2-1
*Physical examination*	
Head circumference (cm)	40.0
Chest circumference (cm)	41.0
Height (cm)	69.0
Weight (kg)	7.0
Temperature (°C)	37.2
Heart rate (beats per minute)	134.0
Respiration rate (times per minute)	32.0
*Biochemical examination*	
Albumin (g/L)	25.0–33.7^*^
Alanine aminotransferase, ALT (IU/L)	77.0–293.0
Glutamate oxalacetate transaminase, GOT (IU/L)	203.0–623.0
Direct bilirubin (umol/L)	24.4–265.0
Total bilirubin (umol/L)	213.5–340.8
Alpha fetal protein (ng/mL)	29,090–121,000
Lactic acid (mmol/L)	2.1–5.4
Glucose (mmol/L)	1.44–4.10
Ammonia (umol/L)	80.0–130.0
*Coagulation test*	
D-dimer (mg/L)	0.49–20.0
Activated partial thromboplastin time (s)	> 180
Thrombin time (s)	23.0–42.8
Prothrombin time (s)	39.2–75.9
Fibrinogen (g/L)	0.6
*Blood routine examination*	
Hemoglobin (g/L)	111.0–115.0
Red blood cell count (per liter)	3.2–3.9 × 10^12^
White blood cell count (per liter)	14.3 × 10^9^
Lymphocytes (%)	38.7–52.5
Neutrophils (%)	35.4–50.0
Platelets count (per liter)	146–479 × 10^9^
*Blood gas analysis*	
pH	7.45
pCO_2_ (mmHg)	32.80
pO_2_ (mmHg)	43.60

^*^Multiple test results are presented as (25.0–29.7).

Biochemical examination revealed hepatic dysfunction, lactic acidosis, and hypoglycemia. The serum levels of albumin, alanine aminotransferase, glutamate oxalacetate transaminase, direct bilirubin, total bilirubin, lactic acid, and glucose were 25.0–33.7 g/L (normal range: 60–83 g/L), 77.0–293.0 IU/L (normal range: 0–40 IU/L), 203.0–623.0 IU/L (normal range: 0–40 IU/L), 24.4–265.0 umol/L (normal range: 0–6 umol/L range), 213.5–340.8 umol/L (normal range: 5.1–17.1 umol/L), 2.1–5.4 mmol/L (normal range: <1.8 mmol/L), and 1.44–4.10 mmol/L (normal range: 3.9–5.8 mmol/L), respectively (Table 1). Biochemical tests also showed extremely high alpha fetal protein 29,090–121,000 ng/mL (normal range: 0 -77 ng/mL) and elevated ammonia 80.0–130.0 umol/L (normal range: 10–47 umol/L). Coagulation tests showed dysfunctional blood clotting. The D-dimer, activated partial thromboplastin time, thrombin time, prothrombin time, and fibrinogen were 0.49–20.0 mg/L (normal range: 0.0–0.3 mg/L), >180 s (normal range: 28.0–44.5 s), 23.0–42.8 s (normal range: 14.0–21.0), 39.2–75.9 s (normal range: 12.0–14.8 s) and 0.6 g/L (normal range: 2.0 -4.0 g/L), respectively (Table 1). Blood gas analysis showed that pH, pCO2, and pO2 were 7.45 (normal range: 7.35–7.45), 32.80 mmHg (normal range: 35.0–45.0 mmHg), and 43.60 mmHg (normal range: 80 -100), respectively (Table 1).

**Figure 1:**
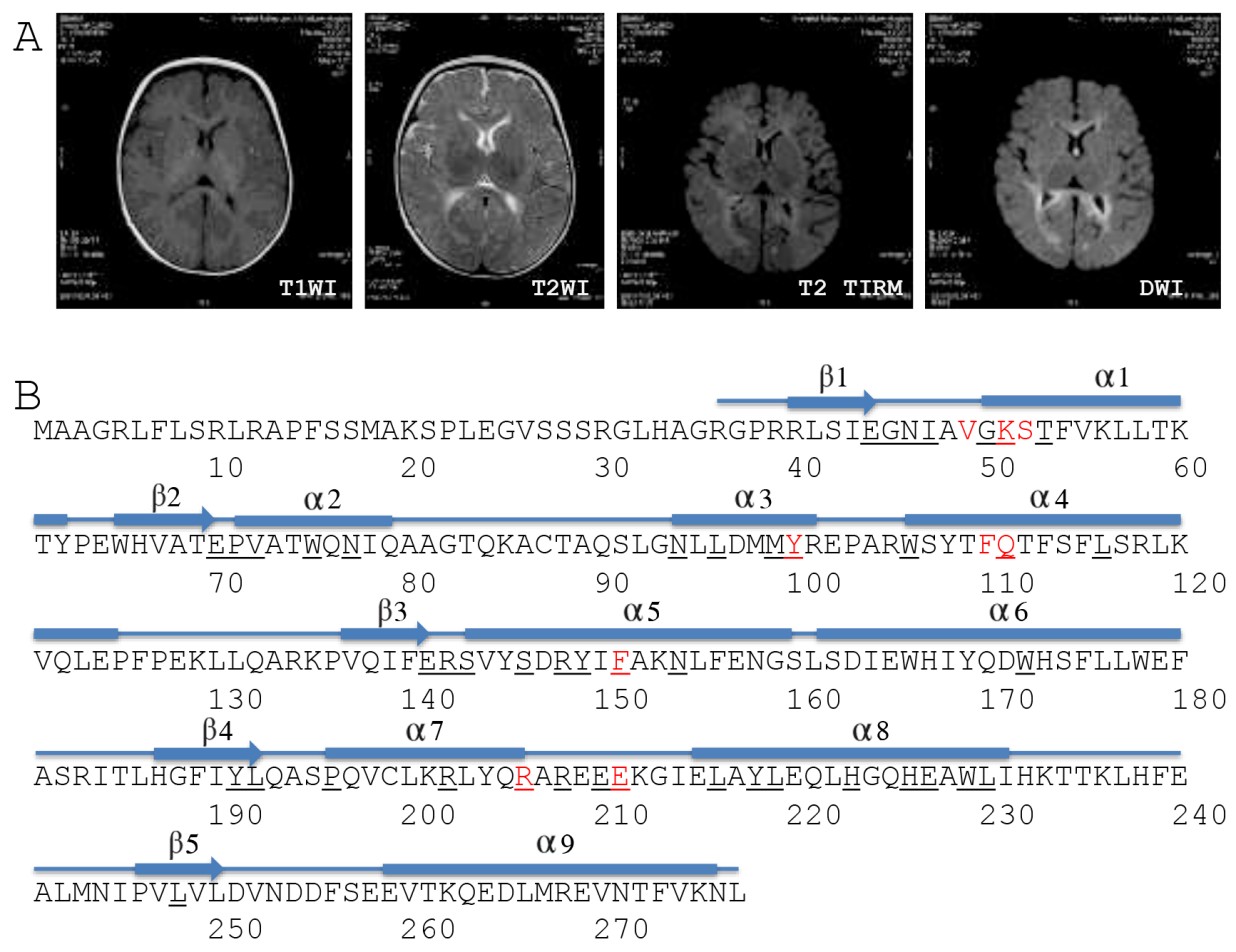
Brain MRI and DGUOK structure **(A)** Brain MRI. T1WI, T1 weighted image; T2WI, T2 weighted image; TIRM, turbo inversion recovery magnitude; DWI, diffusion-weighted magnetic resonance imaging. **(B)** Structure-based sequence alignment of the human DGUOK gene (SwissProt Q16854). The numbers refer to the DGUOK amino acid sequence. Red characters refer to the conserved substrate-binding site. Black fields represent residues identical to the sequences of deoxycytidine kinase, thymidine kinase 2, and deoxyribonucleoside kinase.

Abdominal ultrasonography showed multiple focal liver lesions, hepatic fibrosis, and massive ascites. Brain magnetic resonance imaging (MRI) showed higher DWI and T2WI signals in the bilateral frontal, temporal, parietal, and occipital lobes, the corpus callosum, internal and external capsules, and the brain stem (Figure 1A). In addition, a high T2WI signal also was observed in the bilateral globus pallidus (Figure 1A). Proton magnetic resonance spectroscopic imaging showed normal N-acetylaspartate, choline and creatine peaks in the basal ganglia and thalamus. The results suggested that the patient had hepatocerebral MDS and was thus given symptomatic and supportive treatment for eight days in our hospital. The girl died one and a half months after release from the hospital.

### Genetic findings

Genetic testing revealed that this girl was a compound heterozygote for DGUOK gene alleles c.128T>C (p.I43T) and c.313C>T (p.R105^*^) (Table 2). Her father was a heterozygous carrier of allele c.128T>C (p.I43T). c.128T>C (p.I43T), a novel variant located in exon 1 (National Center for Biotechnology Information ID: NM_080916) that encoded an amino acid in the first beta sheet of the DGUOK protein (Table 2 and Figure 1B). c.128T>C (p.I43T) has been included in the Human Gene Mutation Database, the NHLBI GO Exome Sequencing Project, or the 1000 Genomes Project to date, and has not been previously reported (Table 2). The patient's mother was a heterozygous carrier of allele c.313C>T (p.R105^*^). c.313C>T (p.R105^*^) is located in exon 2 (NM_080916) and encodes amino acids located in the third and fourth alpha helixes of the DGUOK protein (Table 2 and Figure 1B). c.313C>T (p.R105^*^) is predicted to result in a 173 amino acid residue truncation at the C terminus of the DGUOK protein [18]. No prior cases of MDS were found in the family histories of either parent.

**Table 2:** Cumulative frequencies of reported variants of DGUOK gene

Ethnicity / Nationality	Variants	Zygotic type	Sex	Consanguinity	Source	Death	Reference
*DGUOK mutations in children with MDS*							
Chinese	c.128T>C(p.I43T)/c.313C>T(p.R105^*^)	CH	F	No	HP	Yes	this report
French?	c.2T>C(p.M1T)	Homozygote	F	No	HP	Yes	4
French?	c.2T>C(p.M1T)	Homozygote	F	No	HP	Yes	5
Italian?	c.2T>C(p.M1T)/c.677A>G (p.H226R)	CH	M	?	?	?	6
Polish?	c.3G>A(p.M1I)/c.813_814insTTT(p.N271_T272insF)	CH	F	?	?	Yes	7
Polish?	c.3G>A(p.M1I)	Homozygote	F	?	?	Yes	7
Turkish	c.34C>T(p.R12X)	Homozygote	?	Yes	?	Yes	8
Hispanic	c.80delC/c.763_c766dupGATT	CH	F	No	?	Yes	9
Turkish?	c.130G>A(p.E44K)	Homozygote	M2	Yes	HP	Yes	11
French?	c.137A>G(p.N46S)	Homozygote	M	No	?	?	5
American?	c.137A>G(p.N46S)/c.352C>T(p.R118C)	CH	M	No	HP	Yes	13
American?	c.137A>G(p.N46S)/c.352C>T(p.R118C)	CH	M2	No	HP	4y (2009)	13
Hispanic	c.137A>G(p.N46S)/c.352C>T(p.R118C)	CH	M	?	?	Yes	9
Japanese	g.11692_12026del335(p.A48fsX90)	Homozygote	F2	No	?	Yes	14
Japanese	g.11692_12026del335(p.A48fsX90)	Homozygote	F	No	?	Yes	14
German	c.155C>T(p.S52F)	Homozygote	M	Yes	?	1y (2006)	3
Russian	c.155C>T(p.S52F)/c.681-684delGTTT	CH	F2	No	?	Yes	3
European	c.165G>A(p.W65X)/c.487_c490dupGACA	CH	M2	?	?	Yes	9
Israeli-Druze	c.204del A	Homozygote	M5F15	Yes	HP	19 died	15
Arabs	c.223T>A(p.W75R)	Homozygote	F	?	?	Yes	16
Lebanese	c.235C>T(p.79^*^)	Homozygote	F2	Yes	?	Yes	9,17
German	c.313C>T (p.R105^*^)	Homozygote	M	No	HP	Yes	18
French?	c.313 C>T (p.R105^*^)	Homozygote	M	No	?	Yes	5
Indian	c.318 G>A (p.W106^*^)	?	F	No	?	Yes	9,17
European	c.352C>T(p.R118C)	Homozygote	F	No	MUD	Yes	19
Italian	c.444-11C>G/c.605_c.606delGA	CH	M	?	?	Yes	9
Turkish?	c.493G>A(p.E165K)	Homozygote	M	Yes	?	Yes	11
Polish?	c.494A>T(p.E165V)	Homozygote	M	?	?	Yes	7
French?	c.495A>T(p.E165V)/P246R	CH	M	No	?	?	5
Turkish	c.509A>G(p.Q170R)	Homozygote	F	No	HP	2y (2006)	3
French?	c.591A>G/nt424_425delAG	CH	M	No	?	?	5
Hispanic	c.533G>A(p.W178X)	Homozygote	F	No	PUI	Yes	9
American?	c.533G4A (p.W178X)	Homozygote	?	No	PUI	Yes	21
African American	c.572A>G(p.Y191C)	Homozygote	F	No	HP	Yes	22
Chinese	c.572A>G(p.Y191C)/c.151A>C(p.K51Q)	CH	F	?	?	Yes	9
French	c.4G>T(p.A2S)/c.591G>A	CH	M	No	HP	Yes	23
Caucasian/Hispanic	c.605_c606 delGA/c.591G>A (p.Q197Q)	CH	F	?	?	Yes	9
Mexican	c.592-4_c.592-3delTT	Homozygote	F	Yes	HP	Yes	24
Mexican	c.592-4_c.592-3delTT	Homozygote	F	Yes	HP	?	24
Italian?	c.delGA603_604(p.K201fs214X)	Homozygote	M	?	?	Yes	6
American?	c.609_c.610delGT	Homozygote	F2	?	?	Yes	20
Arabs	c. 617G>A(p.R206K)	Homozygote	F	Yes	HP	Yes	16
Hispanic	c.677A>G(p.H226R)/c.592-4_c.592-3delTT	CH	M	?	?	Yes	9
Caucasian	c.677A>G (p.H226R)	Homozygote	M1F1	Yes	?	Yes	25
American?	c.707+417(intron 5)/c.834 (end of 3’UTR)+3416, del3127bp	Homozygote	F	?	?	Yes	26
French?	c.721_724insTGAT+	Homozygote	M	?	?	Yes	5
Italian?	c.749T>C(p.L250S)	Homozygote	F	?	?	Yes	6
French?	c.749T>C(p.L250S)	Homozygote	M1F1	Yes	HP	Yes	4
Italian	c.749T>C(p.L250S)	Homozygote	F	No	?	Yes	27
French?	c.749T>C(p.L250S)	Homozygote	F	Yes	?	?	5
Portuguese?	c.749T>C(p.L250S)	Homozygote	M	Yes	?	Yes	25
Portuguese	c.749T>C(p.L250S)/c.1A>G(p.M1T)	CH	M/F	No	HP	Yes	3
French?	IVS2nt-2/c.749T>C(p.L250S)	CH	F	?	?	Yes	5
American?	c.763_766dupGATT	Homozygote	F2	Yes	HP	Yes	20
Moroccan	c.763_766dupGATT	Homozygote	F	Yes	?	Yes	28
Moroccan	c.763_766dupGATT	Homozygote	?	?	?	Yes	23
Moroccan	c.763_766dupGATT	Homozygote	M2F1	Yes	HP	Yes	29
Algerian	c.763_766dupGATT	Homozygote	M2	No	HP	Yes	29
Moroccan	c.763_766dupGATT	Homozygote	F	Yes	HP	?	29
Italian?	c.763_766dupGATT/c.130g>A(p.E44K)	CH	F	?	?	10mo (2002)	6
Mennonite	c.763G>T (p.D255Y)	Homozygote	M1F1	?	HP	Yes	30
Arabs	c.766_767insGATT(p.F256^*^)	Homozygote	M2	?	?	Yes	16
Polish?	c.766_767insGATT(p.F256X)	Homozygote	F	?	?	Yes	7
French?	c.633A>G(p.E211G)/c.797T>G(p.L266R)	CH	M	No	?	Yes	4
French?	c.495A>T(p.E165V)/c.797T>G(p.L266R)	CH	M	No	?	Yes	4
French?	c.495A>T(p.E165V)/c.797T>G(p.L266R)	CH	M	No	?	Yes	5
Tunisian	c.444-62C>A	Homozygote	M	Yes	HP	Yes	31
Moroccan	c.444-62C>A	Homozygote	M2	Yes	HP	Yes	31
Turkish?	c.707+3_6delTAAG	Homozygote	F	Yes	HP	Yes	11
*Cases of reversible MDS*							
French	c.137A>G (N46S)/c.797T>G(p.L266R)	CH	M	No	HP	10y (2007)	23
American?	c.81_c.82insCC(p.S28P)/c.4G>T (p.A2S)	CH	M	?	?	21y (2012)	10
Italian	c.319T>C (p.S107P)	Homozygote	M	?	?	3.5y (2007)	9
Hispanic	c.137A>G(p.N46S)/c.352C>T(p.R118C)	CH	M	?	?	3.5y (2007)	9
*Cases of MDS underwent liver transplantation*							
American?	c.425G>A(p.R142K)/c.679G>A(p.E227K)	CH	M	No	HP	5y (2002)	20
Polish	c.1A>G(p.M1V)/c.3G>A(p.M1I)	CH	F	No	?	1y (2006)	3
Mennonite	c.763G>T (p.D255Y)	Homozygote	F	?	HP	3y (2005)	30
*DGUOK mutations in adult patients with mitochondrial DNA multiple deletions*							
Italian?	c.130G>A(p.E44K)/c.462 T>A(p.N154K)	CH	F	?	?	72y	12
Italian?	c.186 C>A(p.Y62^*^)/c.509A>G(p.Q170R)	CH	M	?	?	80y	12
Italian?	c.444-11C>G/c.509A>G(p.Q170R)	CH	F1M1	?	?	46y,48y	12
Italian?	c.605_606delGA(p.R202YfsX12)/c.462T>A(p.N154K)	CH	F	?	?	69y	12
Italian?	c.605_606delGA(p.R202YfsX12)/c.137A4G p.N46S	CH	F	?	?	23y	12

?, unspecified; CH, compound heterozygote; HP, heterozygous parents; PUI, paternal uniparental isodisomy; MUD, maternal uniparental disomy; M5F15, 5 males and 15 females in a single kindred; 72y, 72 years old when data collected; 10mo, 10 months old when data collected; 4y (2009), 4 years old in the published year. Cases with confirmed improvement in the pathological manifestation of MDS were sorted in “Cases of reversible MDS”. Three cases of MDS with liver transplantation were collected together in section of “Cases of MDS underwent liver transplantation”.

### Demographic and genetic characteristics of cumulative variants of the DGUOK gene

To map the DGUOK gene mutations to MDS, we collected all reports published in the NCBI database (Table 2). In total, 112 infantile MDS cases had been reported worldwide by the end of April 2017. The reports consisted of 60 (53.6%) females and 49 (43.8%) males; 92 (82.1%) died before 1 year of age; 13 (11.6%) subjects were alive when the study was published [3, 6, 9, 10, 13, 15, 20, 23, 30]; 47 (42.0%) and 35 (31.3%) cases were consanguineous offspring and non-consanguineous offspring, respectively (Table 2); 81 (72.3%) and 31 (27.7%) were homozygotes and compound heterozygotes, respectively (Table 2); and the parents of 55 (49.1%) cases were heterozygous carriers of related DGUOK variants (Table 2).

Three reviewed MDS cases underwent liver transplantation at age 5, 1, and 3 years old in 2002, 2006, and 2005, respectively (Table 2) [3, 20, 30]. There were four reversible MDS cases, and the pathological manifestation of the four cases was confirmed to be improved [9, 10, 23]. The ages of the patients with reversible cases were 10, 21, 3.5, and 3.5 years old in 2007, 2012, 2007, and 2007, respectively (Table 2). It is difficult to find reversible MDS specific variants in the existing data. Although c.319T>C (p.S107P) was found in an Italian reversible MDS case [9], other variants including c.137A>G (N46S), c.797T>G(p.L266R), c.4G>T (p.A2S), c.137A>G(p.N46S), and c.352C>T(p.R118C) were also found in fatal cases (Table 2).

One previous study has reported DGUOK mutations in adults [12]. The subjects ranged in age from 23 to 80 years old. Clinical presentations were variable, and included mitochondrial myopathy with or without progressive external ophthalmoplegia, recurrent rhabdomyolysis, and adult-onset lower motor neuron syndrome with mild cognitive impairment [12]. The genotypes of six individuals in that study were c.130G>A/c.462T>A, c.186C>A/c.509A>G, c.444–11C>G/c.509A>G, c.605_606delGA/c.462T>A, and c.605_606delGA/c.137A>G (Table 2). As the authors indicated, the mutations identified in adults were associated with an infantile hepatocerebral form of MDS [12]. It is also difficult to find adult mtDNA multiple deletion-specific variants.

Taken together, these studies suggest that DGUOK variants can occur anywhere in the DGUOK gene, and that it remains difficult to find reversible MDS-specific or adult mtDNA multiple deletion-specific variants.

## DISCUSSION

MDS is an autosomal recessive disorder. In this report, we describe a fatal MDS case with novel compound heterozygous variants of the DGUOK gene, c.128T>C(p.I43T)/c.313C>T(p.R105^*^). To date, two additional reports have described the c.313C>T(p.R105^*^) variant [5, 18]. The patients in which these mutations were found were both boys, and one was from Germany and one was suspected to be French. Both boys died before 1 year of age. c.313C>T is predicted to result in a truncated DGUOK protein lacking the 173 amino acid residue at the C terminus. c.128T>C (p.I43T) is a novel variant. Because the girl died before reaching 1 year of age, this allele is likely also lethal. Thus, this finding should be considered during pre-pregnancy checkups.

During the data-collection process, we found that variants of the DGUOK gene were varied. As shown in Table 2, all types of genetic variants could be found in the DGUOK gene. Missense mutations [3–13, 15–23, 25, 27, 30, 31] can change highly conserved amino acids [3, 20, 23], affect start codon recognition by the ribosome [7, 10, 23], substitute a nonpolar neutral amino acid with a polar one [16], result in a premature stop codon [5, 12, 17, 18], affect the initial methionine [3], or induce splicing anomalies [23]. Nucleotide deletions [3, 5, 6, 9, 11, 12, 14, 15, 20, 24, 26] can lead to frameshift mutations, which could result in either a premature termination of translation [3, 12, 15, 20, 26] or aberrant splicing [24]. Nucleotide insertions [5, 7, 10, 16] can either change the polarity of an amino acid [10] or lead to a premature termination [10, 16]. Finally, nucleotide duplications [6, 9, 20, 23, 28–30] can produce a frameshift mutation, a premature stop codon [9, 20, 28], or induce mRNA instability due to nonsense mutations [15, 30].

The DGUOK protein consists of five beta sheets and nine alpha helixes (Figure 1B) [32]. Nine amino acids make up a conserved substrate-binding site, and 45 amino acids are identical to deoxycytidine kinase, thymidine kinase 2, and deoxyribonucleoside kinase (Figure 1B) [32]. Although previous reports have shown that some variants change highly conserved amino acids [3, 4, 9, 16, 20, 23], integrated data show that mutations are not confined to these conserved sites. Many variants occur outside of the conserved region [3–9, 11–13, 19, 21, 25, 27, 30].

Hepatocerebral MDS always leads to hepatic failure and a variable neurological phenotype [1–3]. Our patient manifested jaundice, hepatosplenomegaly, abnormal hepatic function, and multiple abnormal signals in the brain. It is worth mentioning that the alpha-fetoprotein levels in all reports was extremely high [7, 11, 16]. In our patient, levels were as high as 29,090 ng/mL, which may occur due to the liver regeneration process or the early onset of hepatocellular carcinoma.

Since the first report of MDS by Moraes *et al*. in 1991 [1], the number of cases has increased, and now stand at more than 100. There is confusion in the position and nomenclature of MDS-related DGUOK variants, and many studies have either not provided a reference sequence or have adopted different sequence nomenclature. In Table 2, we list the original expression of variants. A pathogenic map of the DGUOK gene needs to be created. Therefore, it is important to follow Human Genome Variation Society (http://varnomen.hgvs.org) recommendations to standardize the location and nomenclature of MDS-related DGUOK variants going forward.

The main limitation to this study is that the patient was critically ill, which prevented us from performing biopsies for complete respiratory chain analysis and mtDNA quantification. Therefore, we were unable to provide any molecular evidence regarding the influence of the c.128T>C(p.I43T) variant on DGUOK expression or activity.

In conclusion, our report shows that diversified DGUOK variants can occur anywhere in the DGUOK gene, and that mutations affect both conserved and non-conserved DGUOK amino acids are associated with fatalities.

## MATERIALS AND METHODS

This study was approved by the Research Ethics Committee of Children's Hospital of Fudan University and was conducted under the Declaration of Helsinki ethical principles for medical research involving human subjects. Informed consent was obtained from the child's parents.

### Patient

A Chinese girl, aged 9 months and 4 days, visited our hospital on February 17, 2016, due to jaundice. The patient was a gravidity 3 and parity 1 (G3P1) infant of unrelated parents. The mother was 31 years old and had three pregnancies, with pregnancy terminations occurring at 1 and 3 months due to fetal growth arrest. The father was 32 years old. The patient was delivered by cesarean section at 37 weeks and 2 days due to a breech position, and had a birth weight of 2,550 g. Eight days after birth, she was hospitalized due to recurrent hypoglycemia (1.0–4.1 mmol/L). Physical and laboratory examinations found jaundice, coagulation disorders, and abnormal liver function. After her release from the hospital, the patient visited multiple hospitals for jaundice, weight stagnation, hepatomegaly, and abnormal liver function. Clinical laboratory and imaging examinations were performed in the corresponding clinical departments in our hospital.

### DGUOK gene detection

During multiple hospitalizations, the patient was screened by whole exome sequencing (WES) and multi-gene panels on several occasions to find risk gene variants. WES was performed by an authorized independent gene testing company (Jinyu, Changsha, China). The testing process is briefly described as follows: WES was initiated by preparing a library using 3 μg purified DNA; enrichment was achieved using the SOLiDoptimized Sure Select All Human Exon Kit (Agilent, Technologies, Santa Clara, CA); sequencing was performed using 5500XL sequencers (Life Technologies, Carlsbad, CA). Quality control parameters were strictly observed throughout the WES workflow, and samples failing quality testing were either restarted completely or restarted from an earlier point. Sequence reads were aligned to hg19 using Lifescope v2.1 software (Life Technologies, Shanghai, China) followed by variant analysis of the aligned sequence. Variants were annotated using a custom analysis pipeline. A minimum of 30×median coverage per sample was required for sufficient data quality.

In our hospital, rare diseases in pediatrics are further screened using an in-house multi-gene panel [33, 34]. The panel included the following 40 genes: ATPase phospholipid transporting 8B1, ATP binding cassette subfamily B member 11, ATP binding cassette subfamily B member 4, tight junction protein 2, bile acid-CoA: amino acid N-acyltransferase, claudin 1, hydroxy-delta-5-steroid dehydrogenase, 3 beta- and steroid delta-isomerase 7, Aldo-Keto reductase family 1 member D1, cytochrome P450 family 7 subfamily B member 1, alpha-methylacyl-CoA racemase, cytochrome P450 family 27 subfamily A member 1, 7-dehydrocholesterol reductase, jagged 1, Notch 2, solute carrier family 25 member 13, DGUOK, MPV17, mitochondrial inner membrane protein, fumarylacetoacetate hydrolase, ATP binding cassette subfamily C member 2, UDP glucuronosyltransferase family 1 member A1, NPC intracellular cholesterol transporter 1, NPC intracellular cholesterol transporter 2, galactose-1-phosphate uridylyltransferase, UDP-galactose-4-epimerase, aldolase fructose-bisphosphate A, aldolase fructose-bisphosphate B, keratin 18, keratin 8, UTP4 small subunit processome component, cystic fibrosis transmembrane conductance regulator, glutamyl-TRNA synthetase 2 mitochondrial, hydroxysteroid 17-beta dehydrogenase 4, lipase A lysosomal acid type, peroxisomal biogenesis factor 1, peroxisomal biogenesis factor 5, POU class 1 homeobox 1, HESX homeobox 1, serpin family A member 1, VPS33B interacting protein, apical-basolateral polarity regulator, Spe-39 homolog, and late endosome and lysosome associated VPS33B. Other than DGUOK, no variants in any of the above genes were found. The DGUOK gene variants were further confirmed by Sanger sequencing using DNA from peripheral blood leukocytes.

### Literature search

To understand the relationships between MDS and DGUOK gene variants, we systematically searched all published studies in PubMed. The latest published data were updated in April 2017. The following terms were used to search titles and abstracts: (mitochondrial DNA depletion syndrome or MDS or OMIM 251880) and (deoxyguanosine kinase or DGUOK or dGK). In total, 31 multi-case and single-case reports were reviewed in this study.
